# Disruption of *Hars2* in Cochlear Hair Cells Causes Progressive Mitochondrial Dysfunction and Hearing Loss in Mice

**DOI:** 10.3389/fncel.2021.804345

**Published:** 2021-12-15

**Authors:** Pengcheng Xu, Longhao Wang, Hu Peng, Huihui Liu, Hongchao Liu, Qingyue Yuan, Yun Lin, Jun Xu, Xiuhong Pang, Hao Wu, Tao Yang

**Affiliations:** ^1^Department of Otolaryngology-Head and Neck Surgery, Shanghai Ninth People’s Hospital, Shanghai Jiao Tong University School of Medicine, Shanghai, China; ^2^Ear Institute, Shanghai Jiao Tong University School of Medicine, Shanghai, China; ^3^Shanghai Key Laboratory of Translational Medicine on Ear and Nose Diseases, Shanghai, China; ^4^Department of Otolaryngology-Head and Neck Surgery, Changzheng Hospital, Second Military Medical University, Shanghai, China; ^5^Department of Otolaryngology-Head and Neck Surgery, Taizhou People’s Hospital, The Fifth Affiliated Hospital of Nantong University, Taizhou, China

**Keywords:** *HARS2*, mitochondrial, apoptosis, hair cells, hearing loss

## Abstract

Mutations in a number of genes encoding mitochondrial aminoacyl-tRNA synthetases lead to non-syndromic and/or syndromic sensorineural hearing loss in humans, while their cellular and physiological pathology in cochlea has rarely been investigated *in vivo*. In this study, we showed that histidyl-tRNA synthetase HARS2, whose deficiency is associated with Perrault syndrome 2 (PRLTS2), is robustly expressed in postnatal mouse cochlea including the outer and inner hair cells. Targeted knockout of *Hars2* in mouse hair cells resulted in delayed onset (P30), rapidly progressive hearing loss similar to the PRLTS2 hearing phenotype. Significant hair cell loss was observed starting from P45 following elevated reactive oxygen species (ROS) level and activated mitochondrial apoptotic pathway. Despite of normal ribbon synapse formation, whole-cell patch clamp of the inner hair cells revealed reduced calcium influx and compromised sustained synaptic exocytosis prior to the hair cell loss at P30, consistent with the decreased supra-threshold wave I amplitudes of the auditory brainstem response. Starting from P14, increasing proportion of morphologically abnormal mitochondria was observed by transmission electron microscope, exhibiting swelling, deformation, loss of cristae and emergence of large intrinsic vacuoles that are associated with mitochondrial dysfunction. Though the mitochondrial abnormalities are more prominent in inner hair cells, it is the outer hair cells suffering more severe cell loss. Taken together, our results suggest that conditional knockout of *Hars2* in mouse cochlear hair cells leads to accumulating mitochondrial dysfunction and ROS stress, triggers progressive hearing loss highlighted by hair cell synaptopathy and apoptosis, and is differentially perceived by inner and outer hair cells.

## Introduction

Hearing loss is the most common sensory disorder affecting approximately 6.1% of the world population (Davis and Hoffman, 2019), which can be caused by excessive noise exposure (Guo L. et al., 2021), ototoxic drugs (He et al., 2017; Li et al., 2018; Liu et al., 2019c,^2021^; Zhong et al., 2020), aging (Cheng et al., 2019; Zhou et al., 2020; He et al., 2021), genetic factors (Qian et al., 2020; Cheng et al., 2021; Fu et al., 2021a; Lv et al., 2021; Zhang S. et al., 2021), and infections (Han et al., 2020; He et al., 2020; Zhang Y. et al., 2021). To date, more than 100 genes have been identified associating with genetic hearing loss (Hereditary Hearing Loss Homepage; https://hereditaryhearingloss.org/, updated in September 2021), which contribute to more than 50% of congenital hearing loss as well as a significant portion of pediatric-onset hearing loss (Sheffield and Smith, 2019). Autosomal recessive mutations in a number of genes encoding Aminoacyl-tRNA synthetases (ARSs), including *HARS2* (Perrault syndrome 2), *LARS2* (Perrault syndrome 4), *NARS2* (DFNB94), *IARS2* (Cataracts, growth hormone deficiency, sensory neuropathy, sensorineural hearing loss, and skeletal dysplasia), *PARS2* (Developmental and epileptic encephalopathy 75 including deafness), and *KARS* (DFNB89) may lead to a variety of syndromic and non-syndromic hearing loss (Konovalova and Tyynismaa, 2013; Oprescu et al., 2017; Wang et al., 2020). ARSs are a group of nuclear-encoded enzymes that ensure correct translation of the genetic code by conjugating each of the 20 amino acids to their cognate tRNA molecule in the cytoplasm and mitochondria (Oprescu et al., 2017). Deficiency of ARSs, especially the mitochondrial ARSs (mtARSs) often affect tissues with high metabolic demands such as the brain, muscle, and inner ear (Fine et al., 2019). Clinical features associated with mutations in mtARS-encoding genes typically include encephalopathy, leukodystrophy, cardiomyopathy, ovarian dysgenesis, and deafness (Figuccia et al., 2021).

Characterized by enzyme kinetic assays, yeast complementation assays and studies of patient-derived cell cultures, most of the mtARS mutations have been shown to disrupt its aminoacylation activity (Oprescu et al., 2017; Figuccia et al., 2021). Previous study on a *Dars2* conditional knockout mouse showed that DARS2 depletion in heart and skeletal muscle causes severe dysfunction of mitochondrial protein synthesis in both tissues, which activates various stress responses predominantly in the cardiomyocytes (Dogan et al., 2014). In another *Dars2* neuron-specific knockout mouse, the immune and cell stress pathways have been shown to be initiated prior to behavioral dysfunction and cerebral deficits (Nemeth et al., 2020). Studies in zebrafish and rat have shown that inhibition of WARS2 leads to cardiac angiogenesis defects and impaired heart function (Wang et al., 2016). A mouse line harboring hypomorphic p.V117L mutation in *Wars2* displays various pathologies including progressive hearing loss, adipose dysfunction and hypertrophic cardiomyopathy (Agnew et al., 2018). The homozygous mutant mice have been shown to gradually lose their outer hair cells and spiral ganglion neurons, but the cellular pathogenesis associated with the hearing loss has not been studied in depth.

Numerous studies have shown that mitochondrial dysfunction is an important step toward a broad spectrum of sensorineural hearing loss associated with noise, ototoxic-drug, and aging (Fischel-Ghodsian et al., 2004; Zhang L. et al., 2021). For example, it has been reported that noise exposure leads to mitochondrial swollen and cristae disruption in outer hair cells and stria vascularis in guinea pig and rat (Spoendlin, 1971; Yu et al., 2014), administration of gentamicin induces opening of the mitochondrial permeability transition pore and reduction of mitochondrial membrane potential (Dehne et al., 2002), and accumulation of mitochondrial DNA common deletion contributes to development of age-related hearing loss (Du et al., 2012; Natarajan et al., 2020). As a major source of reactive oxygen species (ROS) production, mitochondrial dysfunction often leads to ROS formation and accumulation after noise (Wu et al., 2020) or ototoxic drug exposure (Fu et al., 2021b), and ultimately lead to death of hair cells and spiral ganglion neurons (Bock and Tait, 2020).

Though mutations in many mtARS-encoding genes are associated with sensorineural hearing loss, their cellular and physiological pathology in inner ear has rarely been investigated *in vivo*. In this study, we established a hair cell specific knockout mouse model for the mitochondrial histidyl-tRNA synthetase encoding gene *Hars2*, whose recessive mutations lead to decreased levels of aminoacylated tRNA*^His^* and sensorineural hearing loss with female ovarian dysgenesis (Perrault syndrome 2) in humans (Pierce et al., 2011), and whose overexpression restores mitochondrial dysfunction caused by a deafness-associated m.12201T > C mutation in tRNA*^His^* (Gong et al., 2020). Morphological and electrophysiological studies of this conditional knockout (CKO) mouse model furthered our understanding of the pathogenic mechanism underlying the mtARS-associated hearing loss.

## Materials and Methods

### Generation and Genotyping of the *Hars2* Conditional Knockout Mice

**FIGURE 1:**
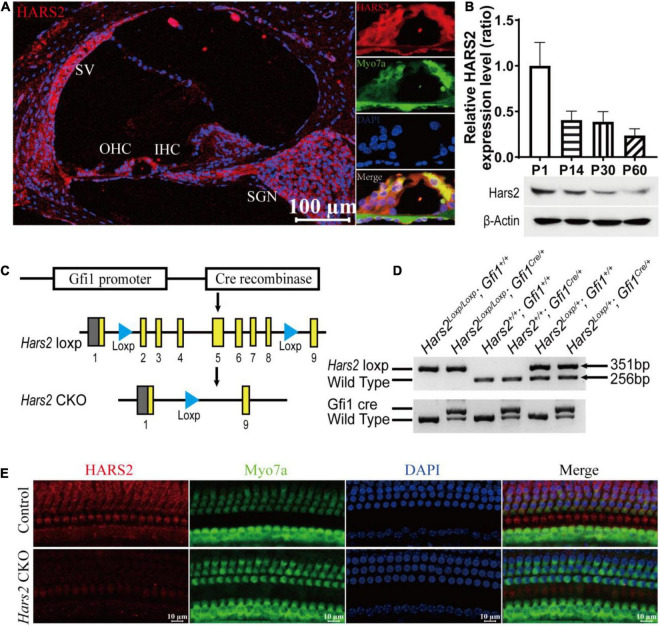
Cochlear expression of HARS2 in the wild-type and *Hars2* CKO mice. **(A)** Immunofluorescence staining of HARS2 in cochlea (left) and organ of corti (right) of the wild-type mice at P30 using anti-HARS2 (red) and anti-Myo7a (green, labeling hair cells) antibodies and DAPI (blue). Scale bar = 100 μm. OHC, outer hair cell; IHC, inner hair cell; SGN, spiral ganglion neuron; SV, stria vascularis. **(B)** Relative protein expression levels of HARS2 at different ages by Western blotting, which was normalized by endogenous β-actin expression. **(C)** Generation of the hair cell-specific *Hars2* CKO mice with the Loxp sites inserted into intron 1 and 8 of mouse *Hars2* and crossed with the *Gfi1^Cre^*. **(D)** Verification of targeted genomic deletion of the *Hars2*-Loxp allele by PCR amplification. **(E)** Immunofluorescence staining of HARS2 in the cochleae of P30 control and *Hars2* CKO mice, showing the successful knockout of *Hars2* in hair cells. Scale bars = 10 μm.

The *Hars2^Loxp/+^* mice were generated using the CRISPR/Cas9 system (GemPharmatech, China). Briefly, Cas9 mRNA, single guide RNAs and donor were co-injected into the zygotes, directing Cas9 endonuclease cleavage and Loxp site insertion in intron 1 and intron 8 of mouse *Har2* (NM_080636.2, Figure 1C). As the *Hars2^Loxp/Loxp^*; ACTB*^Cre/+^* full-body knockout (KO) mice die perinatally, the *Hars2*^Loxp/Loxp^*;Gfi1^Cre/+^* mice (*Hars2* CKO mice) were used to specifically knockout *Hars2* in hair cells (Yang et al., 2010; He et al., 2019). Considering the *Gfi1^Cre/+^* mice have been previously reported to have early-onset, progressive hearing loss (Matern et al., 2017), we used littermates of the *Hars2* CKO and wild-type (WT) control mice under the same *Gfi1^Cre/+^* background. All animal procedures were approved by the Committee of Laboratory Animals of the Ninth People’s Hospital, Shanghai Jiao Tong University School of Medicine. All efforts were practiced to minimize the number of animals used and to prevent their suffering.

For genotyping of the *Hars2* CKO mice, DNA was extracted from mouse tails and PCR amplified using the primers listed in Supplementary Table 1. The PCR products of 351, 256, and 672 bp represent the *Hars2* CKO, wild-type, and *Gfi1^Cre^* alleles, respectively (Figure 1D).

### Auditory Brainstem Response

Auditory brainstem response (ABR) analysis was performed in anesthetized mice at P21, P30, P45, and P60. Following a previously described protocol with minor modification (Zheng et al., 1999; Liu et al., 2019a), hearing thresholds at sound frequencies 4, 5.6, 8, 11.3, 16, 22.6, and 32 kHz were assessed in TDT system 3 (Tucker-Davies Technologies, United States). Briefly, Animals were deeply anesthetized with an intraperitoneal injection of 0.01 g/ml pentobarbital sodium (100 mg/kg), and body temperature was kept on 37°C in the recording process using a Homeothermic Monitoring System (Harvard Apparatus) in a soundproof chamber. ABR was recorded with three subcutaneously implanted stainless needle electrodes. The active electrode was positioned at the vertex, the reference at the right mastoid region, and the ground at the left shoulder. Before ABR analysis was conducted, mice with normal external auditory canal and tympanic membrane were examined using an otoscope. Pure tone bursts were produced by the RZ6 workstation (Tucker-Davis Technologies, United States). Free-field acoustic stimulation was performed through an MF1 speaker (Tucker-Davis Technologies, United States), located 10 cm from the vertex. The evoked potentials were filtered with a bandpass filter from 100 to 3000 Hz and averaged 400 times. For each analyzed frequency, the sound level was reduced from 90 to 0 dB SPL in 5 dB steps. ABR thresholds were determined by minimal stimulus level that evoked any visible recording of waveforms at each frequency. If the hearing threshold could not be measured at 90 dB SPL, the result was noted as threshold of 100 dB SPL for statistical analysis. All amplitudes and latencies of ABR peak I were measured and analyzed using BioSigRZ software (Tucker-Davis Technologies, United States) as previously described (Scimemi et al., 2014; McKay et al., 2015). Amplitude was calculated from the average value of △V on both sides of peak I, and latency referred to the time from the beginning of the stimulus signal to peak I.

### Immunofluorescence Staining and Confocal Imaging

After animals were deeply anesthetized and sacrificed, cochleae were quickly harvested from dissected temporal bones in cold 4% paraformaldehyde (PFA). A small hole was punctured at the top of cochlea and then 4% PFA was slowly perfused through the small hole, oval window and round window. The cochleae were kept in this fixation overnight at 4°C. The next day, after decalcified in 10% EDTA for 2–24 h at room temperature according to postnatal age, the organ of corti was dissected and cut into three turns (apex, middle, and base) for immunofluorescent staining (Akil and Lustig, 2013). For frozen cochlear sections, after making sure the cochleae were soft enough after decalcification, the samples were dehydrated through the 15% and 30% sucrose solution successively for 24 h, immerged into the OCT compound (Sakura Finetek United States, Inc., 4583) and hardened at –20°C. Slices with thickness of 10 μm were cut through the modiolus by a freezing microtome (Leica Biosystems Inc., CM3050S). Slices containing 3–4 organs of corti were collected on glass slides.

For immunofluorescent staining, all samples were first permeabilized with 1% Triton X-100 for 1 h and immersed in blocking solution containing 10% goat serum, 1% bovine serum albumin, and 1% Triton X-100 in PBS (pH 7.2) for 2 h at room temperature. The samples were then incubated with primary antibodies including mouse monoclonal anti-Myo7a (Developmental Studies Hybridoma Bank, MYO7A 138-1), rabbit polyclonal anti-Myo7a (Thermo Fisher Scientific, PA1-936), rabbit polyclonal anti-HARS2 (Abcam, 46545), mouse monoclonal anti-3-nitrotyrosine (3-NT) (Sigma-Aldrich, N5538), rabbit polyclonal anti-4-hydroxynonenal (4-HNE) (Abcam, 46545), and mouse monoclonal anti-CtBP2 (BD Biosciences, 612044) at 1:200 dilution at 4°C overnight. Next day, after three 10-min washing with PBS, the samples were incubated with appropriate secondary antibodies at 1:400 dilution for 2 h at room temperature in darkness, which include Alexa Fluor 488-conjugated goat anti-mouse (CST, 4408S), Alexa Fluor 594-conjugated goat anti-rabbit (CST, 8889S), and Alexa Fluor 488-conjugated goat anti-rabbit (Thermo Fisher Scientific, A32731). According to immunofluorescence staining requirement, the samples were incubated with Alexa-Fluor 647-phalloidin (Thermo Fisher Scientific, A22287) for 1 h at room temperature in darkness. After three final washes with PBS, all immunolabeling samples were mounted on a slide with anti-fade reagent (Invitrogen, P10144). Immunofluorescence images were captured by Zeiss LSM 880 laser confocal microscope (Carl Zeiss Microscopy, Germany).

### Semi-Quantification of the Immunofluorescence Images

Immunofluorescence images for 3-NT and 4-HNE were semi-quantified from original confocal images as previously described (Wu et al., 2020). Briefly, samples were processed in parallel under identical immunofluorescence staining conditions, and confocal microscope images were acquired with the same parameter settings. Using the ImageJ software (NIH, United States), the immunofluorescence images of hair cells were converted into 16-bit grayscale images for further measurement. After subtraction of the background intensity, the averaged grayscale intensity per cell was analyzed. For each repetition, the relative grayscale value was determined by normalizing the ratio to controls.

### Transmission Electron Microscopy

Cochleae were quickly immersed and dissected in 2.5% glutaraldehyde (Sigma-Aldrich, G7651) in phosphate buffer (PB) solution. After fixed with 2.5% glutaraldehyde at 4°C overnight, the basilar membranes were fixed with 1% osmium tetroxide for 2 h at room temperature. The samples were dehydrated through an ethanol and acetone gradient and gradually embedded in Epon-812 (Sigma-Aldrich, 45345). Ultrathin sections were made by a diamond knife on a PowerTome-PC ultramicrotome (RMC, United States) and then placed on copper mesh, sequentially post-stained with uranyl acetate and lead citrate. The samples were imaged with a JEM-1230 transmission electron microscope (JEOL, Japan).

### qPCR Analysis

Cochleae RNA extraction and qPCR analysis were performed as previous described (Vikhe Patil et al., 2015). Briefly, total RNA was extracted from sensory epithelia (without spiral ganglion neurons) of three mice for each genotype group using the TRIzol reagent (Invitrogen, 15596018). cDNA was reverse transcribed by applying RevertAid First Strand cDNA synthesis Kit (Invitrogen, K1622). The primers are listed in Supplementary Table 1. The qPCR was performed on a Roche 480II Real Time PCR System (Roche, United States) using the QuantiNova SYBR Green PCR Kit (QIAGEN, 208052). The mRNA relative expression levels were normalized to the endogenous control *Gapdh*. Each reaction was performed at least in triplicate, and the results were analyzed using the 2^–ΔΔCT^ method (Schmittgen and Livak, 2008).

### Western Blotting Analysis

Five mice (10 cochleae) were sacrificed for each genotype group. The cochleae were rapidly dissected in ice-cold PBS. Tissues of sensory epithelia (without spiral ganglion neurons) were gathered in tubes and mixed with ice-cold RIPA lysis buffer plus protease inhibitor cocktail and phosphatase inhibitors. The samples were homogenized and centrifuged at 10000 × *g* at 4°C for 20 min. The supernatants were collected and the protein concentration was measured using the BCA Protein Assay Kit (Beyotime, P0010). With 5 × SDS sample loading buffer added, the samples were boiled for 5 min and centrifuged at 3000 × *g* for 1 min. A total of 20 μg of each protein sample was separated by polyacrylamide gel electrophoresis (PAGE) and transferred onto a Polyvinylidene Fluoride (PVDF) membrane. The membranes were blocked with 5% non-fat milk for 2 h at room temperature and then incubated overnight at 4°C with the primary antibodies including anti-cleaved caspase-3 Rabbit mAb (CST, 9664) at 1:700, anti-Bcl2 Rabbit mAb (CST, 3498) at 1:700, anti-Cytochrome C Rabbit mAb (CST, 11940) at 1:700, anti-cleaved caspase-9 (CST, 9509) at 1:700, and anti-HARS2 (proteintech, 11301-1-AP) at 1:1000. The membranes were washed three times in TBS with Tween 20 buffer and then incubated with secondary antibody conjugated with horseradish peroxidase for 2 h at room temperature. The immunoreactive bands were detected using a Tanon-4600 Chemiluminescent Imaging System (Tanon, China). The ImageJ software was used to calculate the relative density of probe protein. For HARS2 protein expression analysis, the relative protein expression was determined by normalizing the ratio to P1 HARS2 protein expression.

### Whole-Cell Patch Clamp Recordings

The apical turn of the basilar membrane of the mouse cochlea was micro dissected in the extracellular solution. Whole-cell Patch clamp recordings were performed using the EPC10/2 amplifier (HEKA Electronics, Germany) with the Patchmaster software (HEKA Electronics, Germany) as described in our previous studies (Lin et al., 2019; Liu et al., 2019a; Zhao et al., 2020). Current-voltage relationships of Ca^2+^ influx in inner hair cells (IHCs) were obtained from current responses to ramp depolarization from -90 to 60 mV, and fitted to the following equation:

$$\text{I}\,\left(\text{V}\right)=\left(\text{V}-{\text{V}}_{\text{rev}}\right)\times \frac{{\text{G}}_{\text{max}}}{1+\text{exp}\,\left(-\left(\text{V}-{\text{V}}_{\text{half}}\right)/{\text{K}}_{\text{slope}}\right)}$$

where V is the command membrane potential, V_rev_ is the reversal potential, G_max_ is the maximum conductance, V_half_ is the half activation potential, and the K_slope_ is steepness of voltage dependence in current activation.

Inner hair cell capacitance measurement (C_m_) were performed with the lock-in feature and the “Sine + DC” method in the software Patchmaster (Liu et al., 2019b). Briefly, a 1 kHz sine wave and 70 mV peak-to-peak magnitude was superposed on the IHC holding potential of –90 mV. The averaged capacitances change before and after the depolarization were calculated to monitor exocytosis from IHCs: △C_m_ = C_m_ (response) – C_m_ (baseline). Ca^2+^ current charge (Q_ca_) was calculated by taking the integral of the leak-subtracted current during depolarization.

### *In situ* Apoptosis Staining

Hair cell apoptosis in the cochlea was evaluated using a terminal deoxynucleotidyl transferase-mediated deoxyuridine triphosphate nick-end labeling (TUNEL) staining kit (*in situ* cell death detection kit, Roche). After hair cells were labeled with anti-Myo7a antibody, the cochlear tissues were incubated with freshly prepared TUNEL working solution at 37°C for 1 h in a humidified chamber away from light. After rinsing in PBS three times, Nuclei were counterstained with a 4′, 6-diamidino-2-phenylindole (DAPI) staining solution (Beyotime, P0131). Nuclei of TUNEL positive cells intensely labeled by green were identified as apoptotic cells. Images were captured on a Zeiss LSM 880 laser confocal scanning microscopy (Carl Zeiss Microscopy, Germany).

### Date Processing and Statistical Analysis

Imaging data processing and statistical analyses were carried out with GraphPad Prism 8.0 and Adobe Illustrator CC 2018. A two-tailed, unpaired Student’s *t*-test with Welch’s correction was used for comparisons between the *Hars2* CKO and control mice. For multiple comparison which involves sound level, frequency and cochlear turn, statistical analysis was performed using two-way ANOVA followed by Bonferroni *post-hoc* test. Data were expressed as mean ± SEM. For all statistical analysis, values were considered statistically significant when *P* < 0.05. In Figures, NS represents *P* > 0.05, * represents *P* < 0.05, ^**^ represents *P* < 0.01, ^***^ represents *P* < 0.001.

## Results

### HARS2 Is Robustly Expressed in Postnatal Mouse Cochlea

To explore the role of HARS2 in hearing function, we first examined the expression pattern of HARS2 in the mouse cochlea. As shown in Figure 1A, cryosection immunostaining of the P30 WT mice indicated that HARS2 is widely expressed in the cochlea including inner (IHC) and outer hair cells (OHC), spiral ganglia neurons, stria vascularis, and supporting cells. Western blot analysis in cochleae of different postnatal ages showed that the expression of HARS2 is highest at P1 and gradually decreases at P14, P30, and P60 (Figure 1B).

### The *Hars2* Conditional Knockout Mice Have Rapidly Progressive Hearing Loss

**FIGURE 2:**
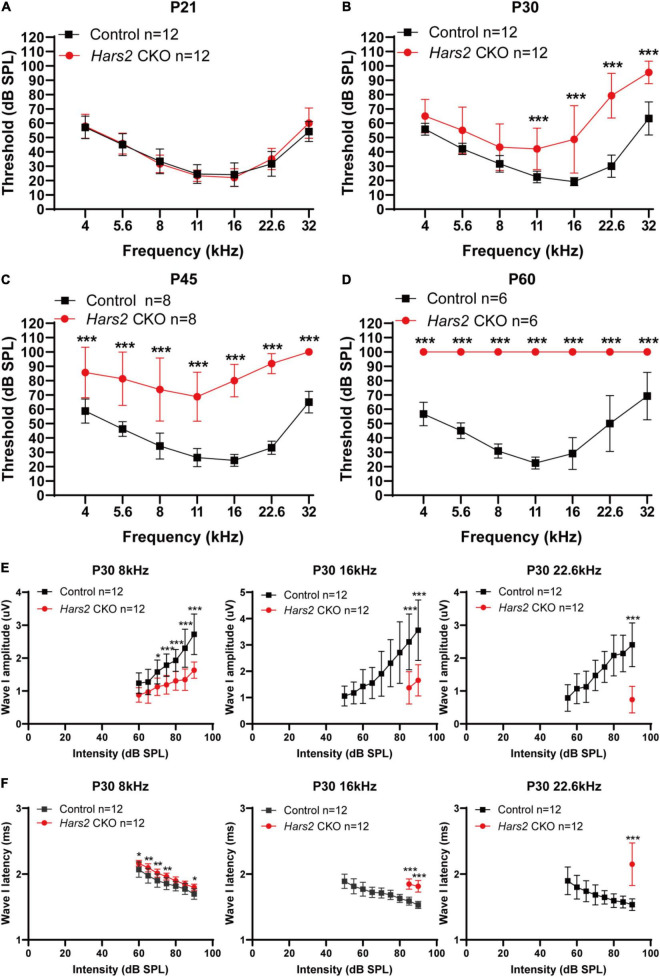
Auditory brainstem response threshold and Wave-I analysis of the *Hars2* CKO mice. **(A)** Indistinguishable ABR thresholds of the *Hars2* CKO and control mice at P21. **(B)** Significantly elevated ABR thresholds at frequencies of 11–32 kHz at P30. **(C)** Further elevated ABR thresholds at all frequencies at P45. **(D)** Profound hearing loss at P60. **(E)** Significantly reduced ABR suprathreshold wave-I amplitudes at frequencies of 8, 16, and 22.6 kHz at P30. **(F)** Significantly prolonged ABR suprathreshold wave-I latencies at frequencies of 8, 16, and 22.6 kHz at P30. * represents *P* < 0.05, ** represents *P* < 0.01, *** represents *P* < 0.001.

The *Hars2^Loxp/Loxp^*;*ACTB^Cre^* full-body KO mice died perinatally, hindering further investigation of *Hars2* in auditory function. To overcome this obstacle, we generated a *Hars2^Loxp/Loxp^*; *Gfi1^Cre^* CKO mouse line (Figures 1C,D). The specifical knockout of *Hars2* in cochlear hair cells was confirmed by immunofluorescence staining (Figure 1E). The *Hars2* CKO mice has apparently normal development. ABR thresholds of the *Hars2* CKO mice were initially indistinguishable from that of the littermate *Hars2^+/+^*; *Gfi1^Cre^* controls at P21 (Figure 2A), but gradually elevated at P30 and P45 and rapidly reached profoundly deaf before P60 (Figures 2B–D). At P30 but not P21, the *Hars2* CKO mice also showed decreased supra-threshold amplitude and prolonged latency of the ABR wave I at sound frequencies 8, 16, and 22.6 kHz (Figures 2E,F and Supplementary Figure 1), which indicates abnormal IHC synaptic transmission, synchronization and auditory nerve conduction (McKay et al., 2015).

### The *Hars2* Conditional Knockout Mice Have Progressive Hair Cell Loss

**FIGURE 3:**
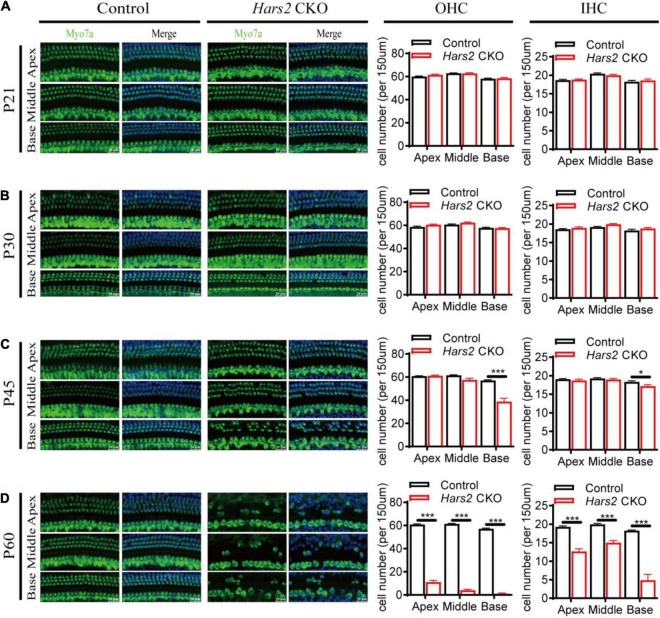
Hair cell loss in the *Hars2* CKO mice. **(A,B)** No significant hair cell loss at P21 and P30. **(C)** Partial loss of hair cells at the basal turn at P45. **(D)** Severe hair cell loss in all turns at P60. Left panels: Whole-mount cochleae were immunostained with anti-Myo7a antibody (green) and DAPI (blue). Right panels: Quantification of the numbers of OHCs (left) and IHCs (right). control: *n* = 5; *Hars2* CKO: *n* = 5. Scale bars = 20 μm. * represents *P* < 0.05, *** represents *P* < 0.001.

Immunostaining of the cochlear hair cells showed that the *Hars2* CKO mice have normal OHC and IHC numbers as the wild-type at P21 and P30 (Figures 3A,B). At P45, mild hair cell loss, more notable in OHCs than IHCs, can be observed at the basal turns of the *Hars2* CKO cochleae (Figure 3C). The hair cell loss proceeds rapidly and extends from the basal turn to the middle and apical. At P60, most OHCs of the *Hars2* CKO mice are lost, especially in the middle and basal turns, while the IHC loss is relatively less severe (Figure 3D).

### *Hars2* Knockout Activates Mitochondrial Apoptosis Pathway in Hair Cells

**FIGURE 4:**
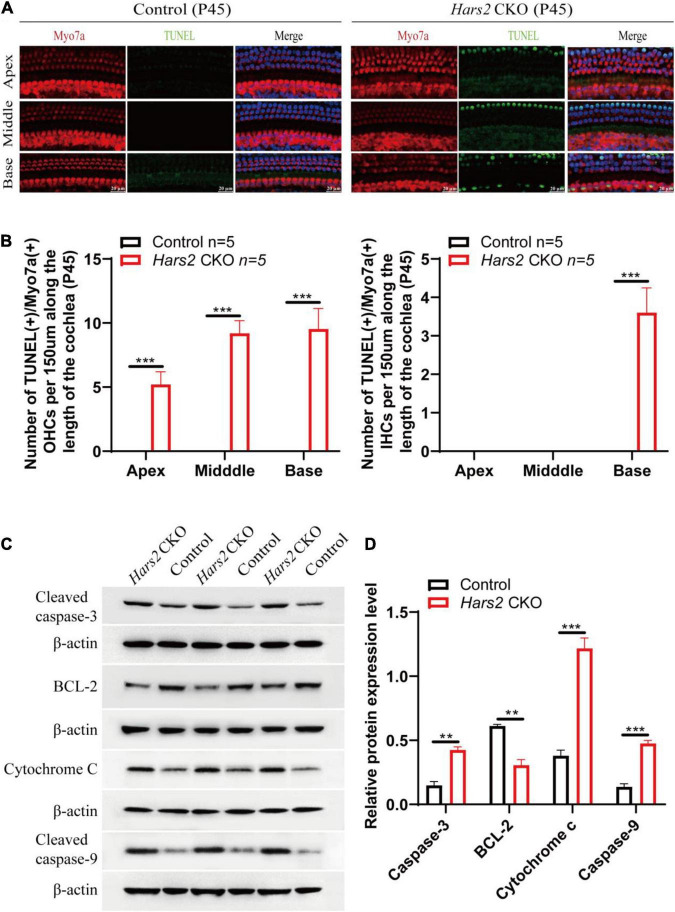
Apoptosis analysis by TUNEL and Western blot in the cochleae of the *Hars2* CKO mice at P45. **(A)** TUNEL (green) and anti-Myo7a (red), DAPI (blue) staining of whole-mount cochlea in the *Hars2* CKO and control mice. Scale bars = 20 μm. **(B)** Quantification of the number of TUNEL positive OHCs and IHCs at the apical, middle, and basal turns of cochlea. **(C)** Protein expression of cleaved caspase-3, BCL-2, cytochrome C, and cleaved caspase-9 detected by Western blotting. **(D)** Relative protein expression levels after normalization against endogenous β-actin. All experiments were replicated in triplets. ** represents *P* < 0.01, *** represents *P* < 0.001.

We next investigated that whether hair cell loss of the *Hars2* CKO mice is due to apoptosis. The TUNEL assay was used to label nuclear DNA fragmentation, a key feature of apoptosis (Majtnerová and Roušar, 2018). At P30, all hair cells remain TUNEL-negative (Supplementary Figure 2). At P45, TUNEL-positive OHCs can be observed in all three turns of the *Hars2* CKO cochleae, while TUNEL-positive IHCs are present in the basal turn only (Figures 4A,B). Expression of multiple pro-apoptotic proteins caspase-3, caspase-9, and cytochrome C are significantly up-regulated in the *Hars2* CKO cochleae, while the anti-apoptotic protein BCL-2 is down-regulated (Figures 4C,D).

### *Hars2* Knockout Elevates Reactive Oxygen Species in Hair Cells

**FIGURE 5:**
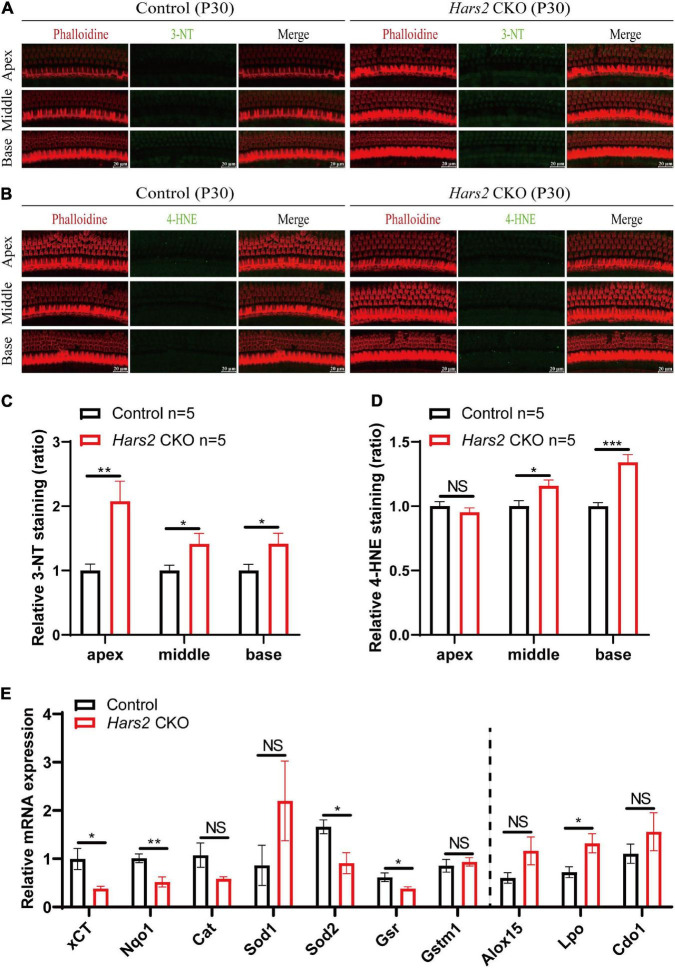
ROS analysis in the cochleae of the *Hars2* CKO mice at P30. **(A,B)** Immunostaining of whole-mount cochleae using anti-3-NT or anti-4-HNE antibodies (green), Phalloidin (red), and DAPI (blue). Scale bars = 20 μm. **(C,D)** Quantification of the 3-NT or 4-HNE immunolabeling in hair cells. **(E)** qPCR analysis of sensory epithelia (without spiral ganglion neurons) showing significantly decreased expression of the antioxidant factors xCT, Nqo1, Sod2, and Gsr and increased expression of pro-oxidant factor Lpo (*n* = 6). NS represents *P* > 0.05, * represents *P* < 0.05, ** represents *P* < 0.01, *** represents *P* < 0.001.

Since mitochondrial ROS has pivotal role in triggering hair cell apoptosis and hearing loss (Wu et al., 2020; Fu et al., 2021b), we then assessed the level of ROS in the *Hars2* CKO cochleae. Increased immunolabeling of the ROS markers 3-NT and 4-HNE can be observed as early as P30 in most turns of cochleae (Figures 5A–D). Quantitative reversed transcribed PCR also showed decreased mRNA expression of several antioxidant enzymes xCT, Nqo1, Sod2, and Gsr, and increased expression of the oxidant enzyme Lpo in the *Hars2* CKO cochleae (Figure 5E). These results suggested that increased ROS in cochlear hair cells is likely the initiating cause for hearing loss in the *Hars2* CKO mice.

### *Hars2* Knockout Disrupts Inner Hair Cell Synaptic Transmission

**FIGURE 6:**
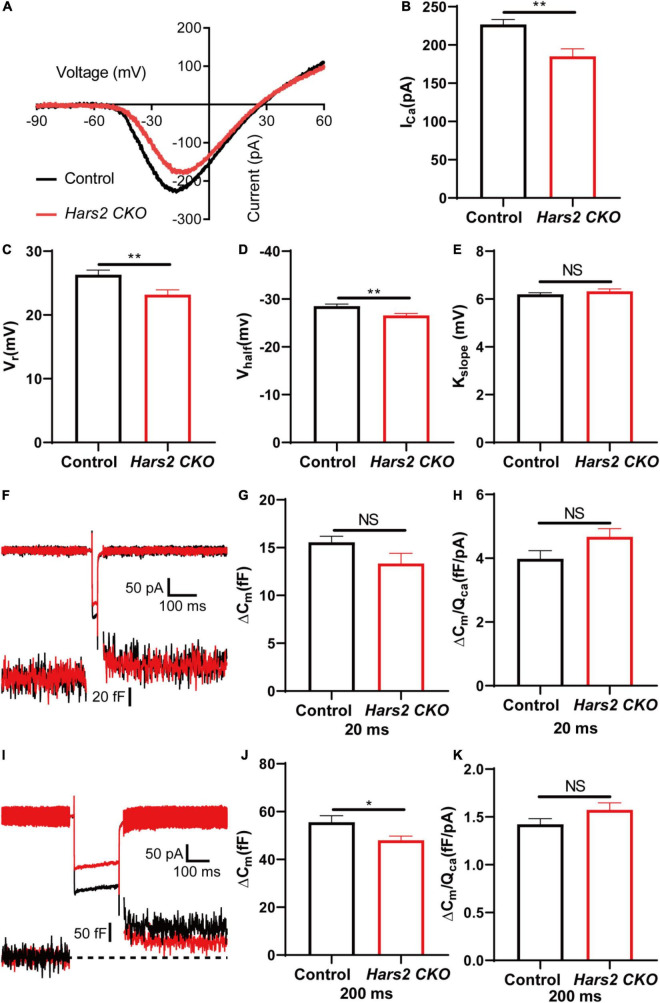
Inner hair cell patch-clamp recording in the *Hars2* CKO mice. **(A)** Representative of the Ca^2+^ current in IHCs of the *Hars2* CKO and control mice. The current response was induced by a voltage ramp from –90 to 60 mV and then leak subtracted. **(B,C)** The Ca^2+^ current amplitude (I_Ca_) and reversal potential (V_rev_) are significantly reduced. **(D)** The half activation potential (V_half_) of calcium current is less negative. **(E)** Slope of activation (k_slope_) is normal. **(F)** Representative traces of Ca^2+^ current (top) and capacitance (bottom) upon 20 ms step depolarization. **(G)** The membrane capacitance change (ΔC_m_) is comparable for stimulations of 20 ms. **(H)** The Ca^2+^ efficiency of exocytosis triggering, quantified as the ratio of ΔC_m_/Q_Ca_, is comparable for stimulation of 20 ms. **(I)** Representative traces of Ca^2+^ current (top) and capacitance (bottom) upon 200 ms step depolarization. **(J)** The ΔC_m_ is significantly reduced for stimulations of 200 ms. **(K)** The ΔC_m_/Q_Ca_ is comparable for stimulation of 200 ms (control: *n* = 5; *Hars2* CKO: *n* = 5). NS represents *P* > 0.05, * represents *P* < 0.05, ** represents *P* < 0.01.

Since the *Hars2* CKO mice has significant hearing loss at P30 without apparent hair cell loss (Figures 2B, 3B), we further investigated the IHC functions of the *Hars2* CKO mice at this age. Whole-mount immunostaining showed that the *Hars2* CKO mice has normal IHC ribbon synapse counts as the controls (Supplementary Figure 3). Whole-cell patch-clamp recording in IHCs, however, recorded a significantly smaller Ca^2+^ current with lower current amplitude (I_Ca_), reversal potential (V_rev_) and half activation potential (V_half_), and normal steepness of voltage dependence (k_slope_) for the *Hars2* CKO mice at physiological conditions (Figures 6A–E). Membrane capacitance change (ΔC_m_), which measures synaptic vesicle release, is normal under short (20 ms) stimulation but significantly reduced under prolonged (200 ms) stimulation (Figures 6F–J). The Ca^2+^ efficiency triggering exocytosis, quantified as the ratio of ΔC_m_/Q_Ca_, remain unaltered for both short and prolonged stimulations (Figures 6H,K). Taken together, these results suggested that the *Hars2* CKO mice have disrupted IHC synaptic transmission due to reduced calcium influx, which likely contributes to the hearing loss at P30 and is consistent with the decreased supra-threshold amplitude and prolonged latency of the ABR wave I (Figures 2E,F).

### *Hars2* Knockout Causes Accumulation of Morphologically Abnormal Mitochondria in Hair Cells

**FIGURE 7:**
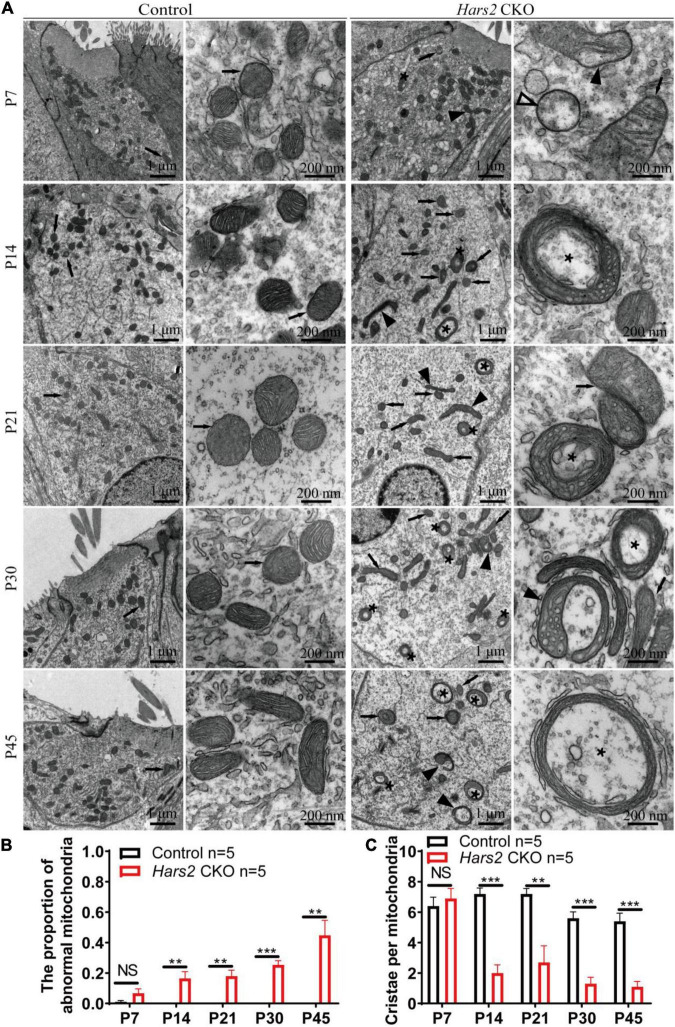
Inner hair cell transmission electron microscopy of the *Hars2* CKO mice between P7 and P45. **(A)** IHCs of the control mice at different ages contains abundant mitochondria with easily-identifiable cristae, whereas various morphologically abnormal mitochondria be observed in IHCs of the *Hars2* CKO mice including swollen (open arrowhead), deformed (black arrowhead) mitochondria, mitochondria with deformed cristae (black arrows), or intrinsic vacuole (black asterisk). **(B)** Quantified proportion of abnormal mitochondria in IHCs. **(C)** Quantified number of mitochondrial cristae per mitochondria in IHCs. NS represents *P* > 0.05, ** represents *P* < 0.01, *** represents *P* < 0.001.

**FIGURE 8:**
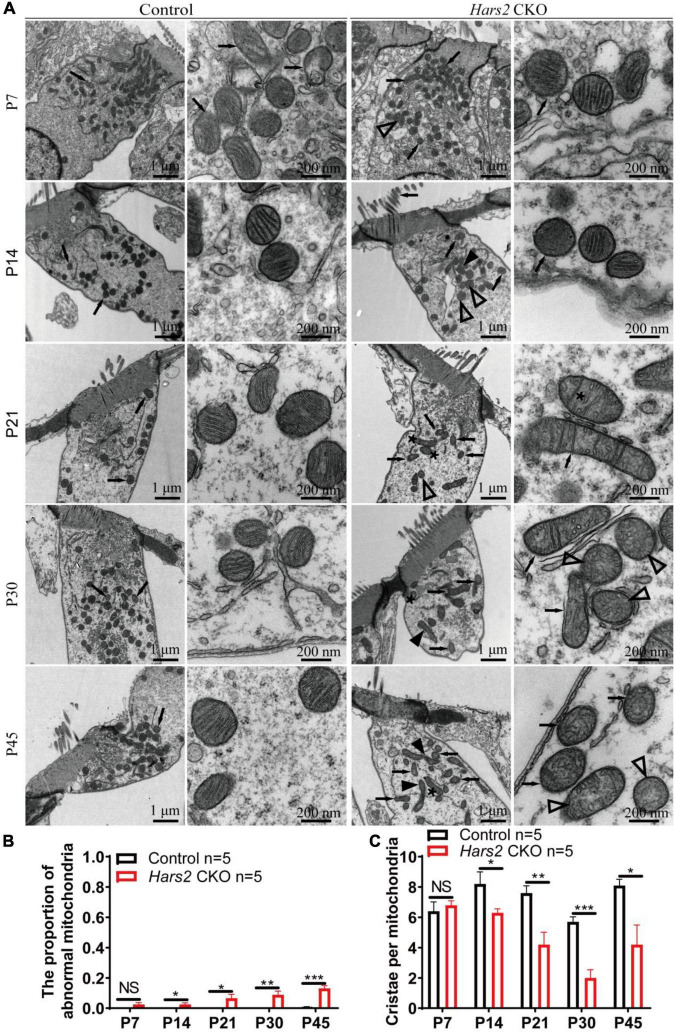
Outer hair cell transmission electron microscopy of the *Hars2* CKO mice between P7-P45. **(A)** OHCs of the control mice at different ages contains abundant mitochondria with easily-identifiable cristae, whereas various morphologically abnormal mitochondria be observed in OHCs of the *Hars2* CKO mice including swollen (open arrowhead), deformed (black arrowhead) mitochondria, mitochondria with deformed cristae (black arrows), or intrinsic vacuole (black asterisk). **(B)** Quantified proportion of abnormal mitochondria in OHCs. **(C)** Quantified number of mitochondrial cristae per mitochondria in OHCs. NS represents *P* > 0.05, * represents *P* < 0.05, ** represents *P* < 0.01, *** represents *P* < 0.001.

We finally evaluated the proportion of morphologically abnormal mitochondria, defined by swelling, deformation, loss of cristae, and emergence of large intrinsic vacuoles, in IHCs (Figure 7) and OHCs (Figure 8) of the *Hars2* CKO mice by Transmission Electron Microscopy (TEM). At P7, the number and distribution of mitochondria in both IHCs and OHCs of the *Hars2* CKO mice are normal, and the ultrastructure of the mitochondria is mostly indistinguishable from the control mice with clearly visible cristae. From P14, a series of abnormal mitochondria can be observed in hair cells of the *Hars2* CKO mice (Figures 7A, 8A), which represents reduced inner membrane surface together with low-density mitochondrial mass. The proportion of morphological abnormal mitochondria appears to increase with age and is larger in IHCs than in OHCs (Figures 7B, 8B). In addition, cristae surface area, positively correlated with the amount of ATP produced by oxidative phosphorylation (van der Laan et al., 2012; Wollweber et al., 2017), significantly decreases in the *Hars2* CKO mice, again more prominently in IHCs than in OHCs (Figures 7C, 8C).

## Discussion

The functions of deafness genes play an essential role on the morphology and development of hair cells (Liu et al., 2019d; Qi et al., 2019, 2020; Chen et al., 2021a), synaptic transmission of spiral ganglion neurons (Guo et al., 2020; Guo R. et al., 2021; Hu et al., 2021; Wei et al., 2021), and many other important components of the inner ear, including supporting cells, greater epithelial ridge cells and lesser epithelial ridge cells (Ding et al., 2020; Zhang et al., 2020; Chen et al., 2021b). In this study, we generated a *Hars2* conditional knockout mice to investigate the function of HARS2 in hearing and its pathogenic mechanism for deafness. Previous studies have shown that missense mutations in *HARS2*, which account for the majority of reported mutations in humans, lead to significantly decreased protein function (Yu et al., 2020), similar to the loss of function effect in this mouse model. Though HARS2 was found to be widely expressed in many cell types of mouse cochlea (Figure 1), the current study chose to focus on cochlear hair cells that have an essential role in converting mechanical sound stimulus into neural electrical signals (Schwander et al., 2010). Notably, our hair cell-specific *Hars2* CKO mice display delayed-onset, rapidly progressive hearing loss (Figure 2), which is very similar to the human hearing phenotype associated with the *HARS2* mutations (Yu et al., 2020), supporting our hypothesis that hair cell is among the primary targets for *Hars2*-related pathogenesis in cochlea. At the same time, we acknowledge that the function of HARS2 in other inner ear cell types, such as spiral ganglion neurons and supporting cells, remain to be further studied.

Underlying increasingly elevated ABR hearing thresholds in postnatal *Hars2* CKO mice (Figure 2), we observed rapidly progressive hair cell loss due to increased ROS level and activated apoptosis pathway (Figures 3, 4, 5). These results are consistent with previous studies on HEK293T cells, which show that the mtARSs are essential in mitochondrial protein synthesis and oxidative phosphorylation (OXPHOS) (Fine et al., 2019; Yu et al., 2020). Reduction of OXPHOS electron transport chain activity may lead to elevated ROS, which in turn can induce opening of mitochondrial permeability transition pore and reduce of mitochondrial membrane potential, triggering mitochondrial apoptotic pathways (Kamogashira et al., 2015).

The hearing loss of the *Hars2* CKO mice, however, cannot be entirely attributed to loss of hair cells, as it already occurs at as early as P30 without apparent hair cell loss (Figures 2B, 3B). In hair cells, neurotransmission of the sound signal relies on rapid and sustained vesicle release of the ribbon synapses, which is an energy demanding process relying heavily on mitochondria (Safieddine et al., 2012). Patch-clamp recordings in acute brainstem slices have demonstrated that energy limitations can negatively affect synaptic transmission (Nagase et al., 2014), with similar results also reported in hippocampal slices (Liotta et al., 2012). In this study, by TEM we observed morphological degeneration of mitochondria, which indicate mitochondrial dysfunction in *Hars2* CKO hearing cells at different postnatal periods including P14. In mammalian hair cells, Ca^2+^ influx current triggers both rapid and sustained exocytosis, which releases the readily releasable pool of synaptic vesicles and ensures their efficient recycling (Meyer et al., 2009; Graydon et al., 2011). Besides energy supply, mitochondria also contributes to maintaining of intracellular calcium homeostasis and influx in hair cells (Wang et al., 2019) and is associated with the susceptibility to noise-induced hearing loss (Liu et al., 2020). Although the number of ribbon synapses in IHCs of the *Hars2* CKO mice is unaltered at P30 (Supplementary Figure 3), our IHC patch-clamp recording showed that the IHC Ca^2+^ influx current is significantly reduced at physiological conditions (Figure 6). Consistently, while the rapid exocytosis and the efficiency of Ca^2+^ triggering exocytosis remains normal, the sustained exocytosis is also reduced in IHCs of the *Hars2* CKO mice. These results are in agreement with previous studies showing that partial block of evoked mitochondria-Ca^2+^ uptake in mature zebrafish hair cells was sufficient to impair presynaptic-Ca^2+^ influx current, especially during sustained stimuli (Castellano-Muñoz and Ricci, 2014; Wong et al., 2019). Overall, our results suggested that *Hars2* deficiency leads to mitochondrial dysfunction, reduced presynaptic Ca^2+^ influx current and compromised sustained exocytosis, which in combination likely contributes to the hearing loss of the *Hars2* CKO mice prior to the hair cell loss.

Our TEM study revealed the progressive morphological abnormalities of mitochondria in hair cells of the *Hars2* CKO mice (Figures 7, 8). Mitochondria is a dynamic organelle whose morphology directly reflects its functional status (Friedman and Nunnari, 2014). Here we calculated the proportion of morphologically abnormal mitochondria and the number of mitochondrial cristae per mitochondria to quantify the mitochondrial dysfunction. Interestingly, the distortion and corruption of mitochondria is far more severe in IHCs than in OHCs of the *Hars2* CKO mice. This is in agreement with previous studies on cisplatin-induced hearing loss and noise-induced hearing loss, which showed similar trends for differential mitochondrial damages between IHCs and OHCs (Hill et al., 2016; Chen et al., 2019). However, both previous and our current studies (Figure 3) showed that OHCs encounter greater cell loss than IHCs under ROS stress (Choung et al., 2009), suggesting that these two hair cell types perceive the mitochondrial damage differently.

**FIGURE 9:**
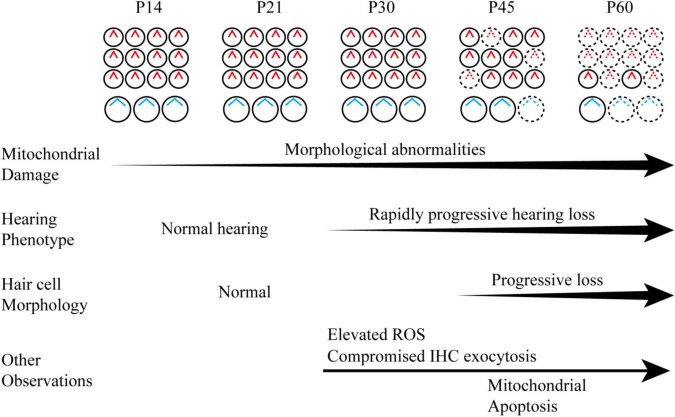
Schematic diagram of the hearing phenotype and hair cell maintenance of the *Hars2* CKO mice between P14 and P60 based on the current morphological and functional studies.

Overall, our study revealed a progressive, rapidly deteriorating course for hair cell mitochondrial damage and hearing loss in the *Hars2* CKO mice, accompanied with elevated ROS, compromised IHC sustained exocytosis and eventual hair cell loss (Figure 9). As antioxidant drugs targeting mitochondrial ROS pathway have been proven effective to relieve hearing loss associated with noise, ototoxic-drug, and aging (Fetoni et al., 2013; Kamogashira et al., 2015; Kim et al., 2019), HARS2 and other mtARSs may present interesting targets for future therapeutic studies.

## Conclusion

Our study suggested that *Hars2* is critically required for hair cell survival and maintenance of appropriate function. Mutations in *Hars2* may lead to progressive, rapidly deteriorating hearing loss by hair cell synaptopathy and mitochondrial apoptosis, which are triggered by accumulating mitochondrial damage and elevated ROS stress.

## Data Availability Statement

The datasets presented in this study can be found in online repositories. The names of the repository/repositories and accession number(s) can be found in the article/Supplementary Material.

## Ethics Statement

The animal study was reviewed and approved by The Committee of Laboratory Animals of the Ninth People’s Hospital, Shanghai Jiao Tong University School of Medicine.

## Author Contributions

TY and HW designed and supervised the whole project. PX, LW, and XP designed, conducted experiments, and analyzed data. HP helped with confocal imaging and TEM observation. HuL and HoL carried out the IHC patch clamp recordings and analyzed data. QY, YL, and JX assisted in generating *Hars2* CKO mice, performed qPCR and WB analysis. XP, TY, and HW acquired funding. PX, XP, and TY wrote and reviewed the manuscript. All authors contributed to the article and approved the submitted version.

## Conflict of Interest

The authors declare that the research was conducted in the absence of any commercial or financial relationships that could be construed as a potential conflict of interest.

## Publisher’s Note

All claims expressed in this article are solely those of the authors and do not necessarily represent those of their affiliated organizations, or those of the publisher, the editors and the reviewers. Any product that may be evaluated in this article, or claim that may be made by its manufacturer, is not guaranteed or endorsed by the publisher.
