# Supplementary material for: Disruption of Hars2 in Cochlear Hair Cells Causes Progressive Mitochondrial Dysfunction and Hearing Loss in Mice
Source: Front Cell Neurosci. 2021 Dec 15;15:804345. doi: 10.3389/fncel.2021.804345 (PMC8715924; doi:10.3389/fncel.2021.804345)
Supplement: Supplementary file 1 [file Data_Sheet_1.docx]

Supplementary Material

# Supplementary Figures and Tables

SUPPLEMENTARY FIGURE 1 | Wave-I analysis and representative ABR wave forms of the *Hars2* CKO mice. (A) Normal ABR wave-I amplitudes and latencies of the *Hars2* CKO mice at P21.(B) Representative ABR wave forms at 16 kHz for WT and *Hars2* CKO mice at P30.

SUPPLEMENTARY FIGURE 2 | TUNEL-positive hair cells are not observed at P30 in the *Hars2* CKO mice.

SUPPLEMENTARY FIGURE 3 | IHC ribbon synapse counts in control and *Hars2* CKO mice at P30. (A) Whole-mount immunostaining of Myo7a (red) and CtBP2 (green) at hair cells corresponding to frequencies of 8, 16 and 22.6 kHz. Scale bars=5 μm. (B) Quantification of CtBP2-positive ribbon synapses.

SUPPLEMENTARY TABLE 1 | The primers sets of PCR and qPCR used in this study.

## Supplementary Figures

## SUPPLEMENTARY FIGURE 1

##
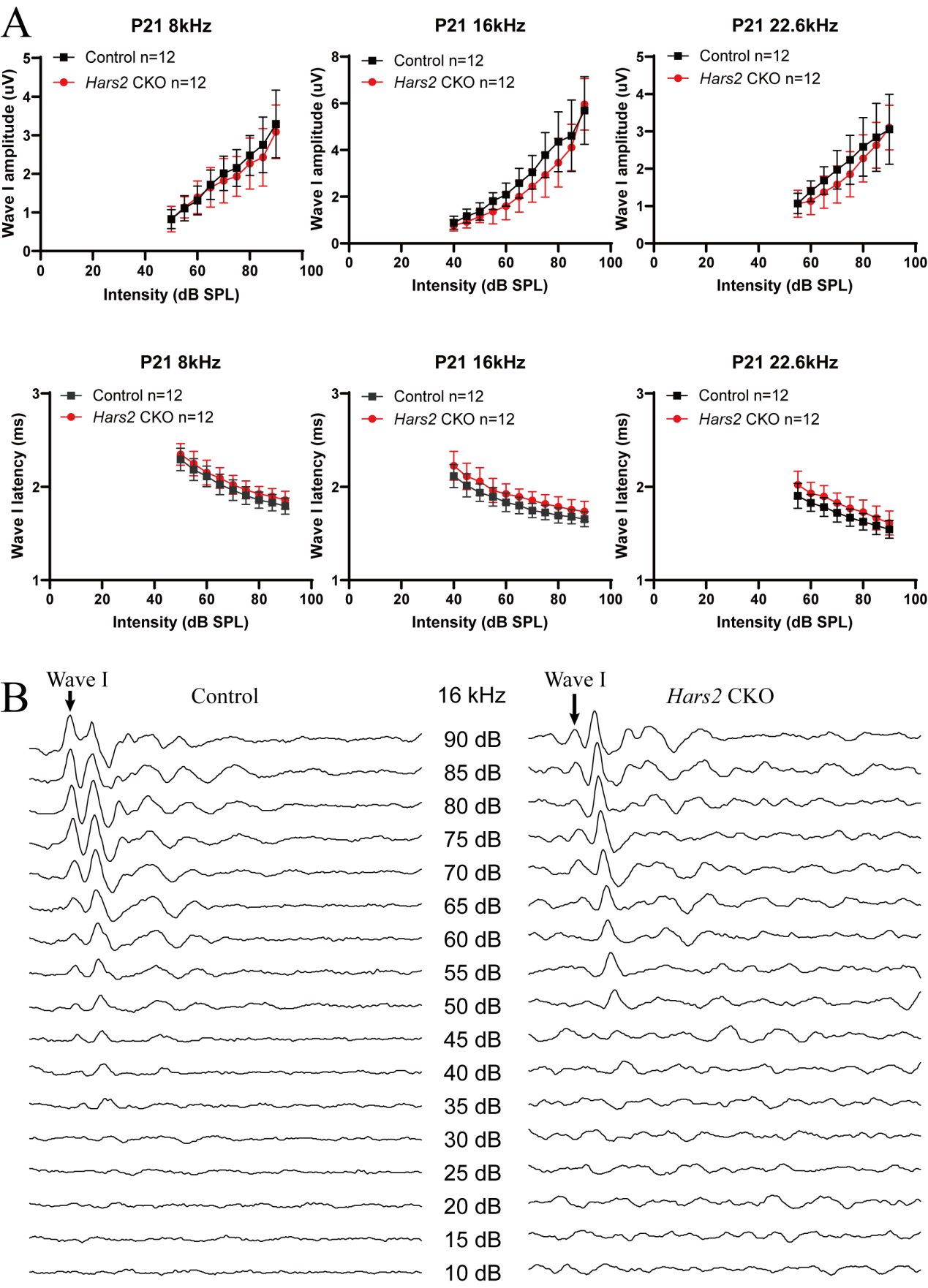


SUPPLEMENTARY FIGURE 2


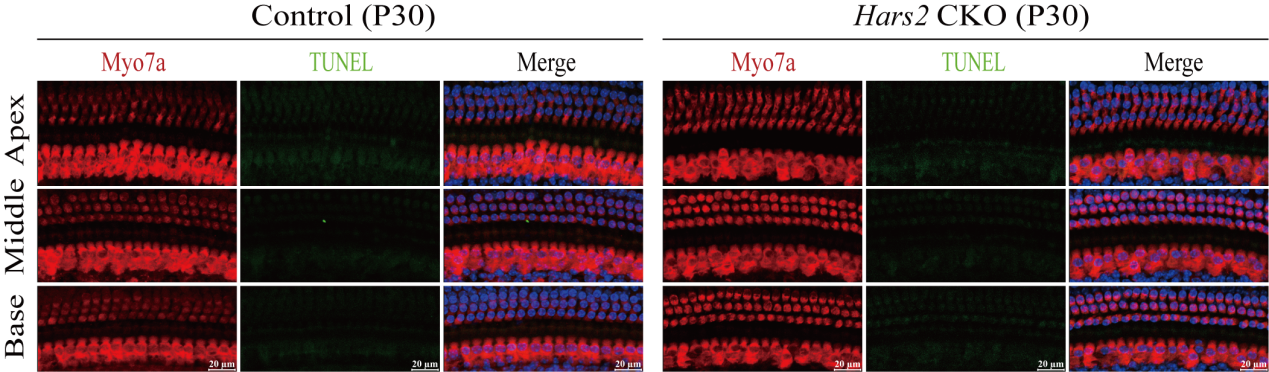


SUPPLEMENTARY FIGURE 3


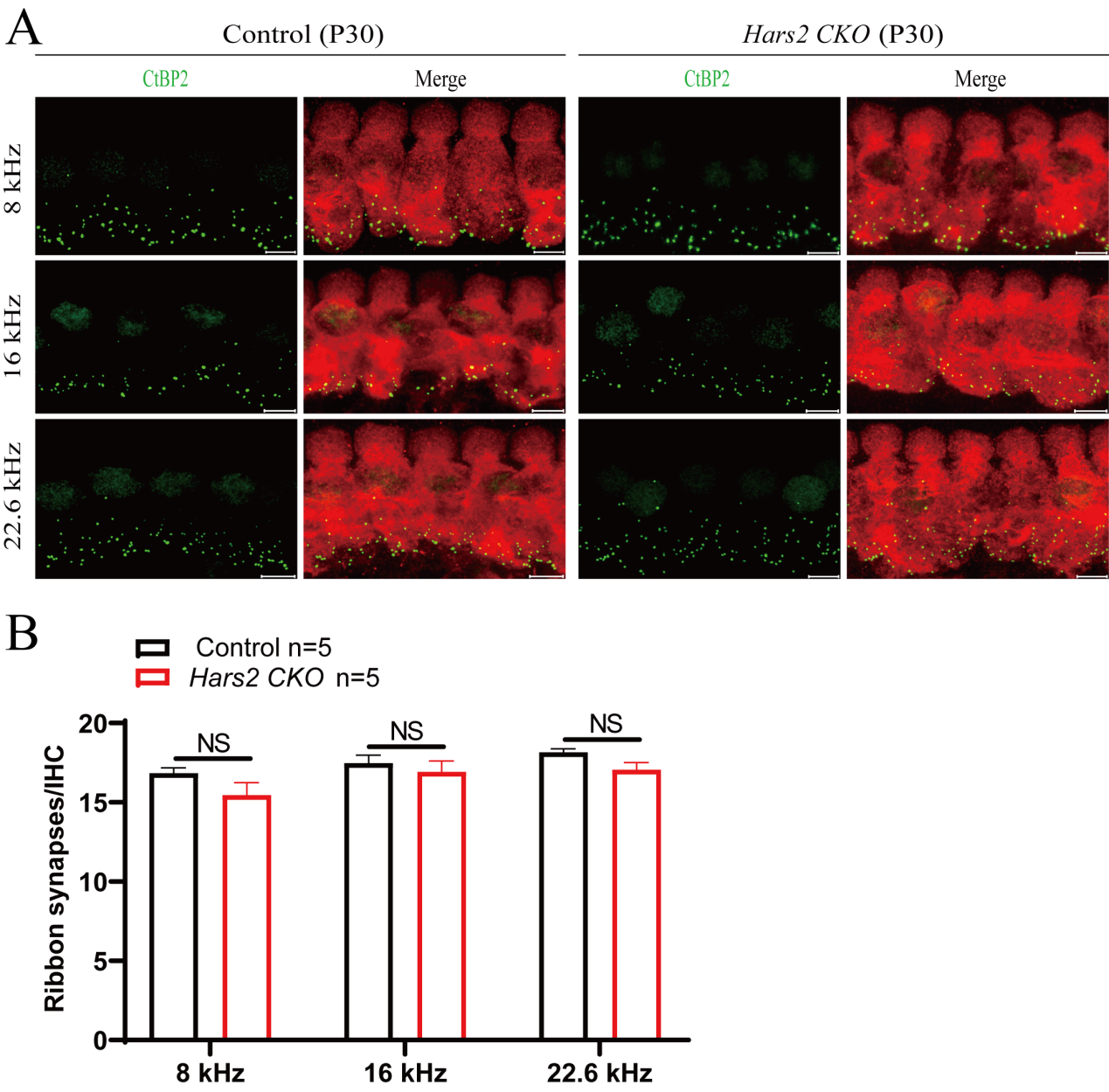


## Supplementary Table

SUPPLEMENTARY TABLE 1

The primers sets of PCR and qPCR used were as follows:

| Gene | Forward primer | Reverse primer |
| --- | --- | --- |
| Hars2 | 5′-GTGTTGGTGCGCTACACAGCAT-3′ | 5′-CACACACACACCTATGGAATGATAGG-3′ |
| Gfi1- wild-type | 5′-GGGATAACGGACCAGTTG-3′ | 5′-CCGAGGGGCGTTAGGATA-3′ |
| Gfi1^Cre^ | 5′-GGGATAACGGACCAGTTG-3′ | 5′-GCCCAAATGTTGCTGGATAGT-3′ |
| xCT | 5’-TGGAGGTCTTTGGTCCTTTG-3’ | 5’-CCAGGATGTAGCGTCCAAAT-3’ |
| Nqo1 | 5’-ACTTCAACCCCATCATTTCCAG-3’ | 5’-TATCACCAGGTCTGCAGCTT-3’ |
| Cat | 5’-AGCGGATTCCTGAGAGAGTG-3’ | 5’-GACTGTGGAGAATCGAACGG-3’ |
| Sod1 | 5’-GGGTTCCACGTCCATCAGTA-3’ | 5’-GGTCTCCAACATGCCTCTCT-3’ |
| Sod2 | 5’-TGTTACAACTCAGGTCGCTCT-3’ | 5’-CTCCCACAGACACGGCTG-3’ |
| Gsr | 5’-TATGTGAGCCGCCTGAACA-3’ | 5’-GTGGCAATCAGGATGTGTGG-3’ |
| Gstm1 | 5’-TCCTGCCCACGTTTCTCTAG-3’ | 5’-AGTCTGTGTATTCCAGGAGCA-3’ |
| Alox15 | 5’-GACTTGGCTGAGCGAGGACT-3’ | 5’-CTTGACACCAGCTCTGCA-3’ |
| Lpo | 5’-CTGGACCAGAAGAGATCCATG-3’ | 5’-TCACCAGGTGGGAACATGATGG-3’ |
| Cdo1 | 5’-GTGGATCAAGGAAATGGA-3’ | 5’-CTTGATCATCTCGTTGGA-3’ |
| Gapdh | 5’-TGCGACTTCAACAGCAACTC-3’ | 5’-CTTGCTCAGTGTCCTTGCTG-3’ |
